# Association of Body Mass Index with Hearing Loss in Korean Adult Population

**DOI:** 10.3390/jpm12050786

**Published:** 2022-05-13

**Authors:** Jong-Seop Koo, So Young Kim

**Affiliations:** 1 CHA University College of Medicine, Seongnam 13496, Korea; jskoo1023@naver.com; 2 Department of Otorhinolaryngology-Head & Neck Surgery, Bundang CHA Medical Center, CHA University, Seongnam 13496, Korea

**Keywords:** hearing, body mass index (BMI), underweight, obesity, age, sex

## Abstract

This study aimed to explore the relationship between body mass index (BMI) and hearing loss. We analyzed data from the Korean National Health Insurance Service Health Screening Cohort 2009–2019 (291,471 patients with hearing loss and 6,088,979 control participants). Both patient groups were subsequently divided into four groups according to BMI: <18.5 (underweight), 18.5–24.9 (normal), 25–29.9 (obese I), and ≥30 (obese II). To evaluate the relationship between BMI and hearing loss, multivariate logistic regression analysis was used, adjusting for age, sex, smoking, alcohol consumption, blood pressure, triglycerides, total cholesterol, low-density lipoprotein, proteinuria, serum creatinine, aspartate aminotransferase, alanine aminotransferase, and fasting glucose levels. The adjusted odds ratio (OR) of the underweight group for hearing loss was 1.21 (95% CI = 1.19–1.24) compared to the normal BMI group, whereas the adjusted ORs of obese I and obese II groups for hearing loss were 0.95 and 0.87, respectively. Being underweight was generally associated with an increased prevalence of hearing loss in the Korean adult population.

## 1. Introduction

In the last few years, over 5% of the world’s population—an estimated 1.57 billion people—has been reported to have hearing loss. Of those, the population of the Western Pacific region accounted for the largest proportion (540.2 million), followed by Southeast Asian population (400 million) [1]. Hearing loss is associated with communication difficulties and a lower quality of life, and it can even lead to depression and social isolation [2]. Various risk factors related to hearing loss have been actively investigated, including hypertension, smoking, diabetes, autoimmune diseases and infections, as well as different etiologies [3,4,5,6,7,8]. Population-based studies from 29 countries conducted between 1973 and 2010 revealed that hearing impairment was positively related to age, and that its prevalence was higher among men (8.4% with a hearing threshold [HT] ≥ 35 decibels Hearing Level [dB HL]) than in women (6.8% HT ≥ 35 dB HL); moreover, hearing impairment was more prevalent in low-income regions, such as sub-Saharan Africa (15.7% HT ≥ 35 dB HL) and South Asia (17.0% HT ≥ 35 dB HL), than in high-income countries (4.9% HT ≥ 35 dB HL) [9].

Obesity constitutes an important public health concern as its prevalence has continued to increase worldwide in the past few decades [10]. Several studies have reported an association between obesity and hearing loss. A recent study showed that patients with a body mass index (BMI) ≥ 27.5 kg/m^2^ had 1.59-fold increased odds of sudden sensorineural hearing loss than those with a BMI < 23.5 kg/m^2^ [11]. In addition, a prospective cohort study of employees aged 20–64 years in Japan found that the adjusted hazard ratios (HRs) for hearing loss at 1 kHz were 1.21 and 1.66 for those with BMI 25.0–29.9 and ≥30.0 kg/m^2^, respectively, compared to individuals with BMI < 25.0 kg/m^2^ [12]. Factors related to obesity that increase the prevalence of sensorineural hearing loss include waist circumference, total cholesterol, triglyceride, BMI, metabolic syndrome, and presence of visceral adipose tissue [13].

However, other studies have reported no relationship between obesity and hearing loss prevalence. A prospective cohort study of male patients aged 40–74 years in the US showed that a history of hypertension (HR 0.96; 95% confidence interval [CI], 0.88–1.03), diabetes mellitus (HR 0.92; 95% CI, 0.78–1.08), or obesity (HR 1.02; 95% CI, 0.90–1.15 for BMI ≥ 30 kg/m^2^ compared to the normal range of 19–24.9 kg/m^2^) was not significantly associated with hearing loss [14]. In order to clarify the association between BMI and severe hearing loss, Kim et al. conducted a cross-sectional study, which reported that severe hearing loss prevalence increased (odds ratio [OR] = 1.312 for BMI ≥ 30 kg/m^2^) or decreased (OR = 1.312 for BMI < 18.5 kg/m^2^) along with BMI. However, the conclusions were limited to severe cases as the study excluded mild to moderate degrees of hearing loss [15].

In summary, being both obese and underweight have been reported to increase the risk of hearing loss. Since the prevalence of obesity is low in Asian populations, the association of obesity with the risk of hearing loss may differ from that in Western populations [16]. In this study, we hypothesized that being underweight or obese increases the risk of hearing loss. The effects of age and sex on hearing loss were analyzed using subgroup analysis.

## 2. Materials and Methods

### 2.1. Study Population

The National Health Insurance Service Health Screening Cohort (NHIS-HEALS) is a cohort of individuals who participated in health screening programs provided by the NHIS in the Republic of Korea. We used data based on the NHIS-HEALS database from 2009 to 2019. The Ethics Committee of CHA Bundang Medical Center (2022-04-009) approved the use of these data. This study was exempted from the need for written informed consent by the Institutional Review Board.

### 2.2. Participant Selection

**Figure 1:**
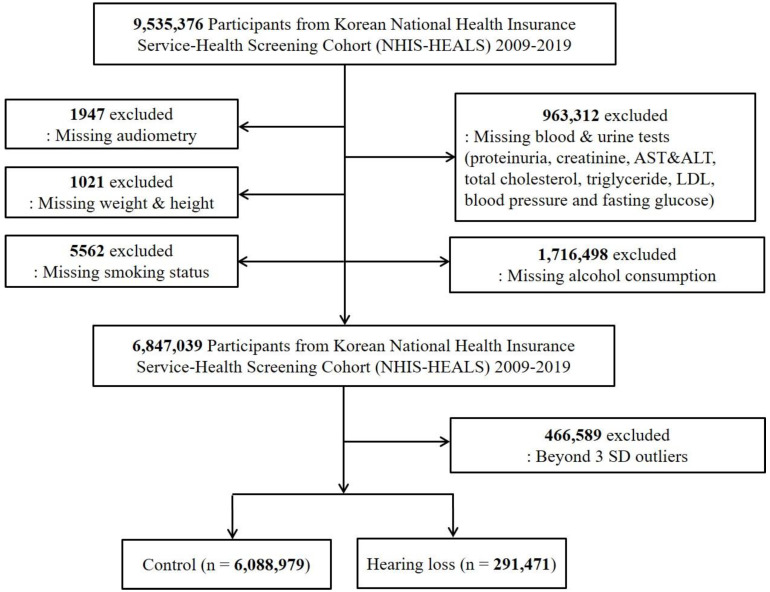
A schematic illustration of the participant selection process used in the present study.

The total number of participants was 9,535,376. Of these, 2,688,340 were excluded because of missing data on hearing loss in one or both ears, smoking, alcohol consumption, height and weight beyond established thresholds, abnormal blood pressure, proteinuria, and abnormal blood levels of creatinine, aspartate aminotransferase (AST), alanine aminotransferase (ALT), total cholesterol, triglycerides, low-density lipoprotein (LDL), and fasting glucose. We detected outliers by determining an interval spanning over the mean plus/minus three standard deviations, as a result, 466,589 were excluded. Consequently, 316,623 participants with hearing loss and 6,530,413 control participants were included in the final analysis (Figure 1).

Of a total of 9,535,376 participants, 316,623 hearing loss patients and 6,530,413 control participants were enrolled.

### 2.3. Variables

Independent variable. BMI (kg/m^2^) was categorized as <18.5 (underweight), 18.5–24.9 (normal), 25–29.9 (obese I), and ≥30 (obese II) [17].

Dependent variable. Hearing loss was defined as an HT > 40 dB HL at 1 kHz using pure-tone audiometry.

Covariates. Age groups were categorized using five-year intervals (40–44, 45–49, 50–54, 55–59, 60–64, 65–69, 70–74, 75–79, 80–84, and ≥85 years). Proteinuria was determined using the dipstick method, which shows a certain degree of color change toward green within 60 s after dipping a test paper into urine. The degree of color change was indicative of protein levels in urine. Proteinuria status was expressed as negative (−), weakly positive (±), 30 mg/dL (+1), 100 mg/dL (+2), 300 mg/dL (+3), and 1000 mg/dL (+4), and categorized into two groups: normal (+1, +2) and abnormal (3+, 4+, 5+, 6+). Creatinine was measured in serum and categorized as <1.4 (normal) and ≥1.4 (abnormal). Smoking status was assessed using a questionnaire and categorized into three groups: current smokers, past smokers, and non-smokers. Alcohol consumption status was categorized into two groups: consumers and non-consumers. Blood pressure was categorized into two groups: systolic blood pressure < 120 mmHg and diastolic blood pressure < 80 mmHg (normal), and systolic blood pressure ≥ 120 mmHg or diastolic blood pressure ≥ 80 (hypertension). AST and ALT blood levels were categorized as ≤40 (normal) and >40 (abnormal). Total cholesterol levels were categorized as <200 (normal) and ≥200 (abnormal). Triglyceride levels were categorized as <200 (normal) and ≥200 (abnormal). LDL levels were categorized as <130 (normal) and ≥130 (abnormal). Fasting glucose levels were categorized as <100 (normal) and ≥100 (abnormal).

### 2.4. Statistical Analyses

A chi-square test was performed to determine the differences in categorical variables between hearing loss and control groups. Multiple logistic regression was used to analyze the crude and adjusted OR for hearing loss. In this analysis, crude (simple) and adjusted models were used. Adjustments were made for age, sex, smoking, alcohol consumption, blood pressure, triglycerides, total cholesterol, LDL, proteinuria, serum creatinine, AST, ALT, and fasting glucose levels.

For subgroup analyses, we subdivided the participants according to age (40–59, 60–79, ≥80 years) and sex. The P value was set at *p* < 0.05. The 95% CIs were calculated. The results were statistically analyzed using SPSS statistical software platform (version 22.0; IBM, Armonk, NY, USA).

## 3. Results

The following analyzed variables—age, sex, smoking, alcohol consumption, BMI, blood pressure, total cholesterol, proteinuria, creatinine, AST, ALT, and fasting glucose levels—were different between the hearing loss and control groups (*p* < 0.001, Table 1). Patients categorized as underweight, normal weight, obese I, and obese II accounted for 3.8%, 63.5%, 29.6%, and 3.0% of the hearing loss group and 3.6%, 64.8%, 28.3%, and 3.3% of the control group, respectively (*p* < 0.001, Table 1).

Compared to the normal BMI group, the underweight group showed a higher odds of hearing loss (adjusted OR = 1.21, 95% CI = 1.19–1.24, *p* < 0.001; Table 2). Conversely, the obese group showed lower odds of hearing loss as the two variables were negatively correlated (adjusted OR = 0.95, 95% CI = 0.95–0.96 for obese I and adjusted OR = 0.87, 95% CI = 0.85–0.89 for obese II, *p* < 0.001; Table 2). Higher triglyceride (adjusted OR = 1.02, 95% CI = 1.01–1.03, *p* = 0.004; Table 2) and LDL (adjusted OR = 1.08, 95% CI = 1.07–1.10, *p* < 0.001; Table 2) levels were associated with an increased odds of hearing loss.

We performed subgroup analyses according to age (40–59, 60–79, >80 years) and sex. Consistent with the results for all age groups (Table 2), men demonstrated proportionally higher odds of hearing loss when being underweight (BMI < 18.5) in all age groups (adjusted OR = 1.34, 95% CI = 1.26–1.42, *p* < 0.001 for 40–59 years old; adjusted OR = 1.58, 95% CI = 1.52–1.65, *p* < 0.001 for 60-79 years old; adjusted OR = 1.20, 95% CI = 1.14–1.26, *p* < 0.001 for ≥80 years old; Table 3), whereas being overweight (BMI ≥ 30) was associated with lower odds of hearing loss (adjusted OR = 0.63, 95% CI = 0.59–0.67, *p* < 0.001 for 40–59 years old; adjusted OR = 0.60, 95% CI = 0.57–0.64, *p* < 0.001 for 60–79 years old; adjusted OR = 0.92, 95% CI = 0.86–0.99, *p* = 0.034 for ≥80 years old; Table 3).

In women, the association of hearing loss with BMI differed according to age (adjusted OR = 0.69, 95% CI = 0.65–0.74, *p* < 0.001 for BMI < 18.5 and 40–59 years old; adjusted OR = 1.11, 95% CI = 1.07–1.14, *p* < 0.001 for 25 ≤ BMI < 29.9 and 40–59 years old; adjusted OR = 1.06, 95% CI = 1.01–1.11, *p* < 0.001 for BMI < 18.5 and 60–79 years old; adjusted OR = 1.11, 95% CI = 1.08–1.13, *p* < 0.001 for for 25 ≤ BMI < 29.9 and 60–79 years old; adjusted OR = 1.31, 95% CI = 1.24–1.38, *p* < 0.001 for for BMI < 18.5 and over 80 years old; Table 3).

## 4. Discussion

The association between BMI and hearing loss in the Korean population was analyzed in this study. Compared to normal BMI (18.5–25 kg/m^2^), lower BMI (<18.5 kg/m^2^) was associated with an increased risk of hearing loss even after adjusting for age, sex, smoking, alcohol consumption, and past medical histories (blood pressure, dyslipidemia, proteinuria, serum creatinine, liver function, diabetes). In the BMI 25–29.9 kg/m^2^ group, although the odds of hearing loss were high in the crude model, they were equivalent to that in the normal BMI group after adjusting for the described variables, indicating that obesity was not related to an increased risk of hearing loss. Our results are partly consistent with those of previous studies in which being underweight was associated with an elevated risk of hearing loss [15,18]. After adjusting for sex, smoking, alcohol consumption, BMI, blood pressure, LDL, triglycerides, total cholesterol, proteinuria, serum creatinine, AST, ALT, and fasting glucose, our results presented strong evidence for an association between being underweight and increased risk of hearing loss.

In women, this study showed that the association of BMI with hearing loss was different according to age. Consistent with this finding, many studies have investigated possible causal factors for the effect of obesity on hearing loss. Adiponectin is released from adipose tissue, increases insulin sensitivity, and is present at low levels in people with obesity. Reduced adiponectin levels have been shown to cause hearing loss, especially at high frequencies; eventually, adiponectin may protect hearing function [19]. Additionally, obesity-induced atherosclerosis may reduce blood flow to the cochlea via stiffening or constriction of the internal auditory artery [20]. In addition, as the levels of free fatty acids and triglycerides increase, oxidative stress and hypoxic damage occur, damaging the capillary wall, which repeats this process [21]. Furthermore, as type 2 diabetes is caused by obesity, the abovementioned process is enhanced because the glucose levels are chronically higher, leading to atrophy of the stria vascularis and loss of cochlear outer hair cells [22,23,24].

Interestingly, being underweight was associated with an increased risk of hearing loss in the current study. According to a previous study, underweight and malnutrition are associated with an increased risk of hearing loss because of extensive degeneration and demyelination of the eighth cranial nerve [25], as well as metabolic lesions, including mitochondrial impairment affecting neurons characterized by high energy consumption [26]. Nutritional factors can affect hearing loss as well [27]. Choi et al. reported that high intake of vitamin A or C combined with high magnesium compared with low intake of both nutrients was significantly associated with lower performances in pure-tone average at high frequencies (214.82%, 95% CI: 220.50–28.74% for β-carotene + magnesium and 210.72%, 95% CI: 216.57–24.45% for vitamin C + magnesium) [28]. They suggested that antioxidative effects may contribute to the suppression of excessive free radical formation in the inner ear, thereby attenuating the risk of hearing loss [28].

Additionally, dyslipidemia was associated with an increased risk of hearing loss in the present study. Several studies have shown that dyslipidemia may reduce hearing function. A cross-sectional study of 40 patients with dyslipidemia showed that higher triglyceride levels were associated with a higher average pure-tone threshold [29]. Suzuki et al. showed that low levels of high-density lipoproteins were associated with atherosclerosis-related microcirculatory disturbances of the cochlear vasculature and increased susceptibility of the cochlea to noise, resulting in an increased risk of hearing loss [30,31].

Combining the above research, our conclusion that low body weight is associated with an increased risk of hearing loss can be explained by introducing the concept of sarcopenic obesity, which describes a condition in which individuals lose their lean mass or skeletal muscle instead of fat [32]. From this point of view, our initial proposal that obesity, not being underweight, was associated with a reduced risk of hearing loss may be conciliated with the present results by attributing BMI ≥ 30 kg/m^2^ groups to metabolically healthy obesity (MHO), which is characterized by normal levels of triglycerides, blood pressure, and fasting glucose, and reduced high-density lipoprotein cholesterol [33]. Both sarcopenic obesity and MHO conditions may be key factors for consideration when reaching a conclusion.

Based on the result of subgroup analyses according to age and sex, being underweight contributed to an increased risk of hearing loss in men of all age group. In the elderly population, being underweight can be partially attributed to malnutrition, which has been suggested to induce high morbidity and mortality [34,35].

This study has several strengths, including a large-scale population-based study with a national health screening cohort. While many studies have previously analyzed the association between obesity and hearing loss, we enrolled patients who were adjusted for many confounders, including age, sex, smoking, alcohol consumption, blood pressure, dyslipidemia, proteinuria, serum creatinine, liver function, and diabetes. However, this study has several limitations. Causality between hearing loss and underweight or obesity cannot be concluded in this cross-sectional study. Despite our efforts to include many confounding factors, there may be residual confounders, such as socioeconomic status, education level, depressive mood, noise exposure, drug use, underlying cardiovascular disease, and congenital hearing loss [36]. In addition, the causes of hearing loss, including maternal rubella or cytomegalovirus infections, intake of ototoxic drugs, and infectious ear disease, could not be identified in this study.

## 5. Conclusions

Being underweight was associated with an increased prevalence of hearing loss. These results were more consistent in men than in women. Since Asians have a lower prevalence of obesity than Western populations, proper weight control, including optimal nutrient intake, is needed from the perspective of hearing loss.

## Figures and Tables

**Table 1:** General Characteristics of Participants. BMI, body mass index; LDL, low-density lipoprotein. * Chi-square test. Significance at *p*-value < 0.05.

Characteristics	Total Participants			Characteristics	Total Participants		
	Hearing Loss (*n*, %)	Control (*n*, %)	*p*-Value		Hearing Loss (*n*, %)	Control (*n*, %)	*p*-Value
Age (years old)			<0.001 *	Blood pressure			<0.001 *
40–44	9817 (3.4)	743,665 (12.2)		Normal	175,078 (60.1)	3,055,041 (50.2)	
45–49	12,762 (7.8)	721,063 (11.8)		Hypertension	116,393 (39.9)	3,033,938 (49.8)	
50–54	22,630 (7.1)	874,311 (14.4)		Triglyceride (≥200)			0.489
55–59	20,687 (7.1)	649,950 (10.7)		Yes	38,159 (13.1)	794,466 (13.0)	
60–64	30,496 (10.5)	821,826 (13.5)		No	253,312 (86.9)	5,294,513 (87.0)	
65–69	28,241 (9.7)	576,966 (9.5)		LDL (≥130)			0.757
70–74	40,639 (13.9)	598,335 (9.8)		Yes	88,427 (30.3)	1,848,939 (30.4)	
75–79	29,262 (10.0)	390,729 (6.4)		No	203,044 (69.7)	4,240,040 (69.6)	
80+	96,937 (33.3)	712,134 (11.7)		Total Cholesterol (≥200)			<0.001 *
				Yes	120,856 (41.5)	2,643,014 (43.4)	
Sex			<0.001 *	No	170,615(58.5)	3,445,965 (56.6)	
Male	158,741 (54.5)	3,148,677 (51.7)		Proteinuria			<0.001 *
Female	132,730 (45.5)	2,940,302 (48.3)		Abnormal	8968 (3.1)	128,000 (2.1)	
Smoking			<0.001 *	Normal	282,503(96.9)	5,960,979 (97.9)	
No	183,935 (63.1)	3,767,870 (61.9)		Creatinine (≥1.4)			<0.001 *
Past	59,447 (20.4)	1,078,171 (17.7)		Yes	5195 (1.8)	40,668 (0.7)	
Active	48,089 (16.5)	1,242,938 (20.4)		No	286,276 (98.2)	6,048,311 (99.3)	
Alcohol consumption			<0.001 *	AST (>40)			<0.001 *
Yes	132,257 (45.5)	2,969,941 (48.8)		Yes	17,431 (6.0)	305,759 (5.1)	
No	159,214 (54.6)	3,119,038 (51.2)		No	274,040 (94.0)	5,783,220 (95.0)	
BMI				ALT (>40)			
<18.5	11,143 (3.8)	221,279 (3.6)		Yes	22,935 (7.9)	578,965 (9.5)	
18.5~24.9	185,217 (63.5)	3,945,739 (64.8)		No	268,536 (9.5)	5,510,014 (90.5)	
25~29.9	86,393 (29.6)	1,720,321 (28.3)		Fasting Glucose (≥100)			<0.001 *
≥30	8718 (3.0)	201,640 (3.3)		Yes	115,459 (39.6)	1,905,076 (31.3)	
				No	176,012 (60.4)	4,183,903 (68.7)	

**Table 2:** Crude and adjusted odds ratios (95% confidence interval) of BMI, Triglyceride, and LDL for hearing loss. * Multiple logistic regression model, Significance at *p* < 0.05. † Adjusted model for age, sex, smoking, alcohol consumption, blood pressure, triglycerides, total cholesterol, LDL, proteinuria, serum creatinine, AST, ALT, and fasting glucose levels, Significance at *p* < 0.05.

Characteristics	Odds Ratio (95% CI)			
	Crude	*p*-Value	Adjusted †	*p*-Value
BMI (kg/m^2^)		<0.001 *		<0.001 †
<18.5 (*n* = 232,422)	1.073 (1.052–1.094)	<0.001 *	1.213 (1.189–1.238)	<0.001 †
18.5~24.9 (*n* = 4,130,956)	1.00		1.00	
25~29.9 (*n* = 1,806,714)	1.070 (1.061–1.079)	<0.001 *	0.954 (0.946–0.962)	<0.001 †
≥30 (*n* = 210,358)	0.921 (0.901–0.941)	<0.001 *	0.873 (0.854–0.893)	<0.001 †
Triglyceride ≥ 200 mg/dL		0.480		<0.001 †
Yes (*n* = 832,625)	1.004 (0.993–1.015)	0.480	1.017 (1.006–1.029)	<0.004 †
No (*n* = 5,547,825)	1.00		1.00	
LDL ≥ 130 mg/dL		0.755		<0.001 †
Yes (*n* = 1,937,366)	0.999 (0.991–1.007)	0.755	1.084 (1.071–1.097)	<0.001 †
No (*n* = 4,443,084)	1.00		1.00	

**Table 3:** Subgroup analysis of crude and adjusted odds ratio (95% confidence interval) of BMI for hearing loss according to age. * Multiple logistic regression model, Significance at *p* < 0.05. † Adjusted model for sex, smoking, alcohol consumption, blood pressure, LDL, triglyceride, total cholesterol, proteinuria, serum creatinine, AST, ALT, fasting glucose, Significance at *p* < 0.05.

Characteristics	Odds Ratio (95% CI)			
	Crude	*p*-Value	Adjusted †	*p*-Value
Male, 40–59 years old, *n* = 1,649,531				
BMI (kg/m^2^)		<0.001 *		<0.001 †
<18.5 (*n* = 38,320)	1.258 (1.185–1.335)	<0.001 *	1.340 (1.262–1.423)	<0.001 †
18.5~24.9 (*n* = 995,271)	1.00		1.00	
25~29.9 (*n* = 558,891)	0.954 (0.934–0.975)	<0.001 *	0.909 (0.889–0.930)	<0.001 †
≥30 (*n* = 57,049)	0.657 (0.615–0.702)	<0.001*	0.627 (0.586–0.670)	<0.001 †
Male, 60–79 years old, *n* = 1,260,410				
BMI (kg/m^2^)		<0.001 *		<0.001 †
<18.5 (*n* = 30,008)	1.645 (1.580–1.713)	<0.001 *	1.582 (1.519–1.648)	<0.001 †
18.5~24.9 (*n* = 749,709)	1.00		1.00	
25~29.9 (*n* = 443,601)	0.843 (0.829–0.857)	<0.001 *	0.858 (0.843–0.872)	<0.001 †
≥30 (*n* = 37,092)	0.547 (0.516–0.580)	<0.001 *	0.603 (0.568–0.639)	<0.001 †
Male, +80 years old, *n* = 397,477				
BMI (kg/m^2^)		<0.001 *		<0.001 †
<18.5 (*n* = 12,744)	1.351 (1.288–1.417)	<0.001 *	1.201 (1.144–1.262)	<0.001 †
18.5~24.9 (*n* = 240,969)	1.00		1.00	
25~29.9 (*n* = 136,278)	0.859 (0.841–0.876)	<0.001 *	0.915 (0.895–0.934)	<0.001 †
≥30 (*n* = 7,486)	0.817 (0.759–0.879)	<0.001 *	0.922 (0.856–0.994)	0.034 †
Female, 40 – 59 years old, *n* = 1,405,354				
BMI (kg/m^2^)		<0.001 *		<0.001 †
<18.5 (*n* = 90,837)	0.590 (0.553–0.629)	<0.001 *	0.691 (0.647–0.737)	<0.001 †
18.5~24.9 (*n* = 1,031,145)	1.00		1.00	
25~29.9 (*n* = 242,702)	1.272 (1.234–1.311)	<0.001 *	1.105 (1.071–1.140)	<0.001 †
≥30 (*n* = 40,670)	1.338 (1.254–1.427)	<0.001 *	1.110 (1.039–1.186)	0.002 †
Female, 60 – 79 years old, *n* = 1,256,084				
BMI (kg/m^2^)		<0.001 *		<0.001 †
<18.5 (*n* = 48,251)	0.970 (0.927–1.015)	0.184	1.061 (1.013–1.110)	0.011 †
18.5~24.9 (*n* = 855,163)	1.00		1.00	
25~29.9 (*n* = 3,030,584)	1.235 (1.212–1.258)	<0.001 *	1.105 (1.084–1.126)	<0.001 †
≥30 (*n* = 49,086)	1.209 (1.161–1.259)	<0.001 *	1.047 (1.005–1.092)	0.029 †
Female, +80 years old, *n* = 411,594				
BMI (kg/m^2^)		<0.001 *		<0.001 †
<18.5 (*n* = 12,26)	1.533 (1.459–1.611)	<0.001 *	1.309 (1.244–1.378)	<0.001 †
18.5~24.9 (*n* = 258,699)	1.00		1.00	
25~29.9 (*n* = 121,658)	1.010 (0.989–1.032)	0.345	1.003 (0.981–1.025)	0.812
≥30 (*n* = 18,975)	1.017 (0.971–1.066)	0.471	0.985 (0.939–1.033)	0.525

## Data Availability

Restrictions apply to the availability of these data. Data were obtained from the Korean National Health Insurance Sharing Service (NHISS) and are available at https://nhiss.nhis.or.kr (accessed on 25 January 2022) with the permission of the NHIS.
